# miR-224-5p and miR-545-5p Levels Relate to Exacerbations and Lung Function in a Pilot Study of X-Linked MicroRNA Expression in Cystic Fibrosis Monocytes

**DOI:** 10.3389/fgene.2021.739311

**Published:** 2021-11-12

**Authors:** Paul J. McKiernan, Kevin P. Molloy, Arlene M. A. Glasgow, Noel G. McElvaney, Catherine M. Greene

**Affiliations:** ^1^ Department of Medicine, Royal College of Surgeons in Ireland, Education and Research Centre, Beaumont Hospital, Dublin, Ireland; ^2^ Department of Clinical Microbiology, Royal College of Surgeons in Ireland, Education and Research Centre, Beaumont Hospital, Dublin, Ireland

**Keywords:** x-linked miRNAs, cystic fibrosis, x chromosome, miR-224-5p, miR-545-5p, Smad4, biomarkers, monocytes

## Abstract

Altered microRNA expression patterns in bronchial brushings from people with versus without cystic fibrosis (CF) relate to functional changes and disease pathophysiology. The expression of microRNAs encoded on the X chromosome is also altered in peripheral blood monocytes of p. Phe508del homozygous versus non-CF individuals. Here we investigate whether levels of the top seven X-linked microRNAs (miR-224-5p, miR-452-5p, miR-450b-5p, miR-542-3p, miR-450a-5p, miR-424-5p, and miR-545-5p) that are significantly increased over 1.5 fold in CF versus non-CF monocytes correlate with lung function. CD14^+^ monocytes were isolated from males and females with (*n* = 12) and without cystic fibrosis (*n* = 12) and examined for the expression of X-linked microRNAs by qRT-PCR array. MicroRNA target mRNA levels were quantified using qRT-PCR. Clinical correlations with lung function data were analysed in the CF cohort. Increasing levels of miR-545-5p correlated moderately with FEV1% predicted (*r* = -0.4553, *p* > 0.05) and strongly with exacerbation rate (*r* = 0.5858, *p* = 0.0483). miR-224-5p levels were significantly higher in the severe (FEV1 <40%) versus mild (FEV1 ≥80%, *p* = 0.0377) or moderate (FEV1 40–79%, *p* = 0.0350) groups. MiR-224-5p expression inversely correlated with lung function (FEV1%: *r* = -0.5944, *p* = 0.0457) and positively correlated with exacerbation rates (*r* = 0.6139, *p* = 0.0370). These data show that peripheral blood monocyte miR-545-5p and miR-224-5p levels correlate with exacerbation rate, whilst miR-224-5p levels also correlate with lung function in cystic fibrosis.

## Introduction

Cystic Fibrosis (CF) is an autosomal recessive disease caused by mutations in the gene encoding cystic fibrosis transmembrane conductance regulator (CFTR), a chloride ion channel. The most prevalent *CFTR* mutation is p. Phe508del, which results in protein misfolding, retention in the endoplasmic reticulum, and consequently reduced functional CFTR at the cell surface. In addition to the primary *CFTR* defect there are other important factors that can contribute to CF lung disease pathology including, for example, intrapulmonary proteases, mucus hypersecretion and microRNA (miRNA) expression in bronchial epithelium (Cantin et al., 1989; Voynow et al., 1998; Voynow et al., 1999; Oglesby et al., 2010), amongst others. MiRNAs are non-coding regulatory RNAs that control protein expression levels. Functionally altered miRNA expression profiles are evident in people with CF and *in vitro* CF cell models (De Santi et al., 2020) and have been reviewed elsewhere (Glasgow et al., 2018).

There is a clinical need to find better strategies for monitoring early lung disease in people with CF in order to identify those at risk for more progressive lung disease and thereby allow earlier intervention (Laguna et al., 2018). The processes of bronchoscopy and bronchoalveolar lavage fluid sampling that are commonly used to determine lung inflammation and damage are highly invasive. Identifying less invasive biomarkers of lung function decline and/or the intrapulmonary inflammatory or infective milieu are highly sought after for CF and other chronic inflammatory lung diseases (Vencken et al., 2015). As fine-tuners of many molecular processes, miRNAs are known to be altered in many disease states, both in cellular expression levels and extracellularly in body fluids such as plasma or sputum (Cui et al., 2019). Given that they can be quantified with high accuracy via qPCR, and offer the prospects of less invasive sampling, miRNAs are attractive candidate biomarkers.

It is commonly recognized that females with CF have worse outcomes than men, for example lower lung function, earlier colonization with respiratory pathogens such as *Pseudomonas aeruginosa*, and greater frequency of exacerbations (Demko et al., 1995; Konstan et al., 2007; Levy et al., 2008). Sex hormones, in particular estrogen, have been implicated in the gender bias of CF lung disease (Chotirmall et al., 2010; Chotirmall et al., 2012; Holtrop et al., 2021). However, there is a lack of studies on the contribution of other major determinants of sex differences, e.g., the sex chromosomes, to CF pathology. The X chromosome is relatively rich in miRNAs, encoding approximately 10% of the total microRNAome. Previously we reported that X chromosome-encoded miRNAs are functionally increased in CF monocytes (McKiernan et al., 2018). Therein miR-224-5p was the most highly increased X–linked miRNA in CF versus non-CF monocytes; its validated target, *SMAD4,* was reciprocally decreased in the same samples. Here we further examined the expression pattern of the mostly highly differentially expressed X-linked miRNAs in monocytes from males and females with and without CF to determine whether they correlate with lung function and exacerbation rates in people with CF.

## Results

### Study Group Demographics

Following informed consent in line with a protocol approved by Beaumont Hospital Ethics Committee (13/108), twenty-four individuals were recruited into this study; 12 were p. Phe508del homozygous individuals (Table 1) confirmed by genotyping, and 12 were non-CF controls, with no underlying lung disease with a mean age of 23.5 ± 5.1 years and 27.3 ± 3.6 years, respectively. Samples were obtained from CF patients at the time of their routine outpatient clinic review (i.e., samples were not from exacerbating inpatients). An exacerbation was defined as worsening of the patients respiratory symptoms requiring treatment with antibiotics. Table 1 shows the CF patient characteristics including infection/colonisation status for pathogens associated with CF lung disease pathophysiology. There were no statistically significant differences between the CF male and CF female cohort in colonisation status for any individual pathogen (Supplementary Figure S1), or total number of pathogens (Supplementary Figure S2). Supplementary Figure S3 shows that forced expiratory volume in 1 s percent predicted (FEV1% predicted) and exacerbation rate (defined as the number of exacerbations in the current year) were not different between the CF males and CF females.

**TABLE 1:** Demographics and colonisation status of the CF cohort.

CF ID	Gender	Age (Years)	*PA* ^a^	Mucoid *PA*	*SA* ^b^	*Asp* ^c^	*Can* ^d^	Others
CFm_01	M	28	+	+	+	-	+	—
CFm_02	M	22	+	-	+	+	+	—
CFm_03	M	27	+	+	+	-	+	MRSA^e^
CFm_04	M	18	-	-	+	-	+	—
CFm_05	M	18	+	-	+	-	+	—
CFm_06	M	24	+	-	+	-	+	Bcc^f^
CFf_01	F	20	+	+	+	-	-	—
CFf_02	F	18	-	-	+	-	+	MRSA, SM^g^
CFf_03	F	21	+	+	+	-	+	—
CFf_04	F	23	+	-	-	+	-	—
CFf_05	F	29	+	+	-	+	+	—
CFf_06	F	34	+	+	-	+	+	—

a *Pseudomonas aeruginosa*.

b *Staphylococcus aureus*.

c *Aspergillus species*.

d *Candida species*.

e llmethicillin-resistant *Staphylococcus aureus*.

f *Burkholderia cepacia* complex.

g *Stenotrophomonas maltophilia*.

### X-Linked miRNA Profiles

**FIGURE 1:**
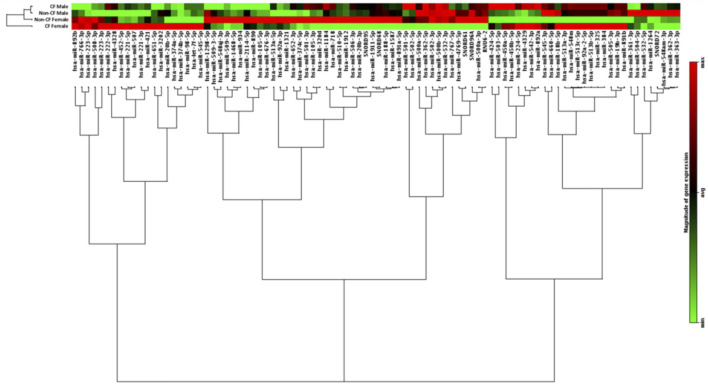
Expression of X-chromosome located miRNA in CD14^+^ monocytes from people with and without CF, annotated by gender and CF pathology. Hierarchical clustering dendrogram showing expression patterns of X-chromosome located microRNAs in males and females with and without CF (*n* = 6 each). CD14^+^ monocytes were obtained from the peripheral blood of each individual. RNA was isolated from each sample and the expression of X-chromosome located miRNAs was examined by qRT-PCR array. Colour density is proportional to magnitude of expression.

Microarray profiling of 86 miRNAs located on the X chromosome was carried out using miScript PCR Array on peripheral blood CD14^+^ monocytes from the CF and non-CF study populations as originally described (McKiernan et al., 2018). Differences in miRNA expression were observed between individuals and when compared by gender and CF pathology, CF females were most distantly related to CF males with respect to the expression of the X-linked miRNAs measured (Figure 1).

### Expression of Some miRNAs Located on the X Chromosome Is Altered in Cystic Fibrosis Versus Non-CF Monocytes

qRT-PCR array data were analysed to determine whether a relationship existed between CF lung disease pathology and the expression of miRNA on the X chromosome in monocytes. Seven X chromosome miRNAs were increased ≥1.5 fold in CF versus non-CF controls. Supplementary Table S1 shows details of the fold-change increase and *p* value for these differentially expressed (DE) miRNAs as first reported (McKiernan et al., 2018). The full microarray profiling data of X-miRNAs in CF versus non-CF monocytes is shown in Supplementary Table S2. No miRNAs were significantly decreased in the CF versus non-CF samples. No miRNAs were differentially expressed ≥1.5 fold between CF males and CF females (other than miR-452-5p which was 1.64-fold higher in CF males), or all males versus all females (data not shown).

### miR-224-5p and miR-545-5p Correlate With Lung Function in People With Cystic Fibrosis

**FIGURE 2:**
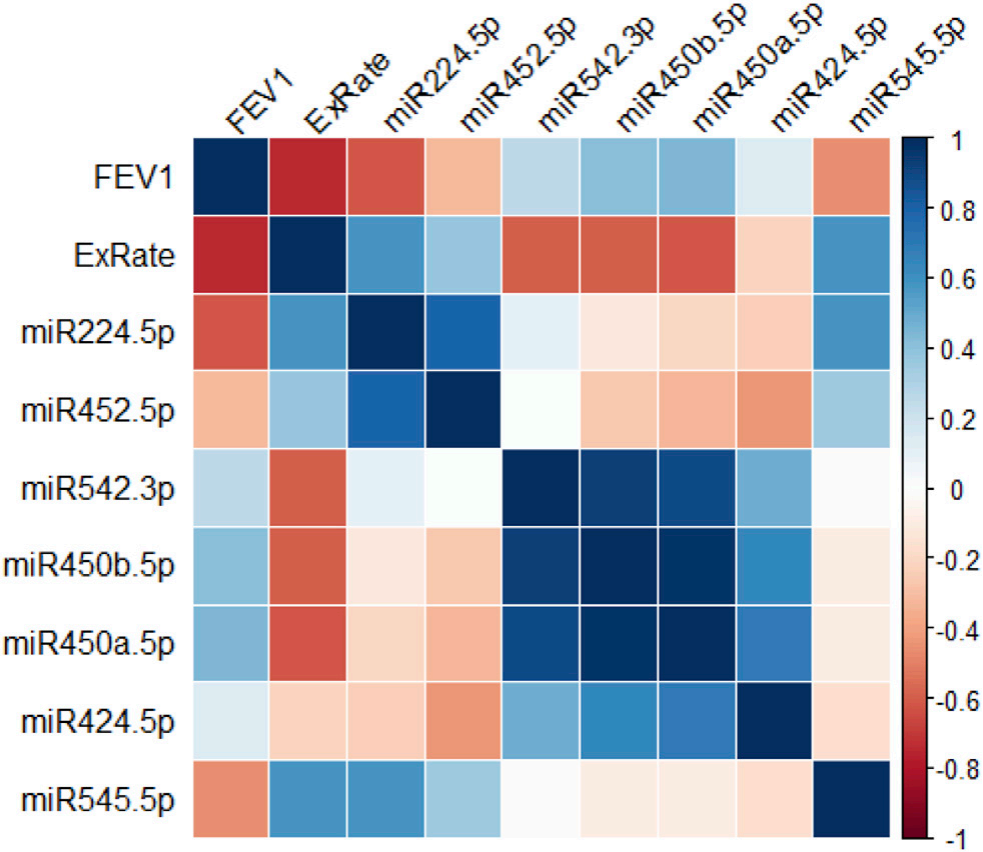
X-linked miRNA expression, lung function and exacerbations. Correlogram of the relationships between levels of top 7 altered X-linked miRNA in peripheral blood monocytes and lung function parameters in people with CF (*n* = 12). *r* values are depicted as a color scale ranging from red (−1) to blue (+1).

The relationship between the DE X-linked miRNA in CF monocytes and lung function data was examined. In this study, due to the small number of samples, we focused on miRNAs with the greatest fold-change difference rather than the lowest *p*-value. Figure 2 is a correlation matrix of lung function (FEV1% predicted, exacerbation rate) and each of these miRNAs. MiR-224-5p shows the strongest inverse correlation with FEV1% predicted, and strongest positive correlation with exacerbation rate. After miR-224-5p, miR-545-5p demonstrates the next strongest, similar pattern.

**FIGURE 3:**
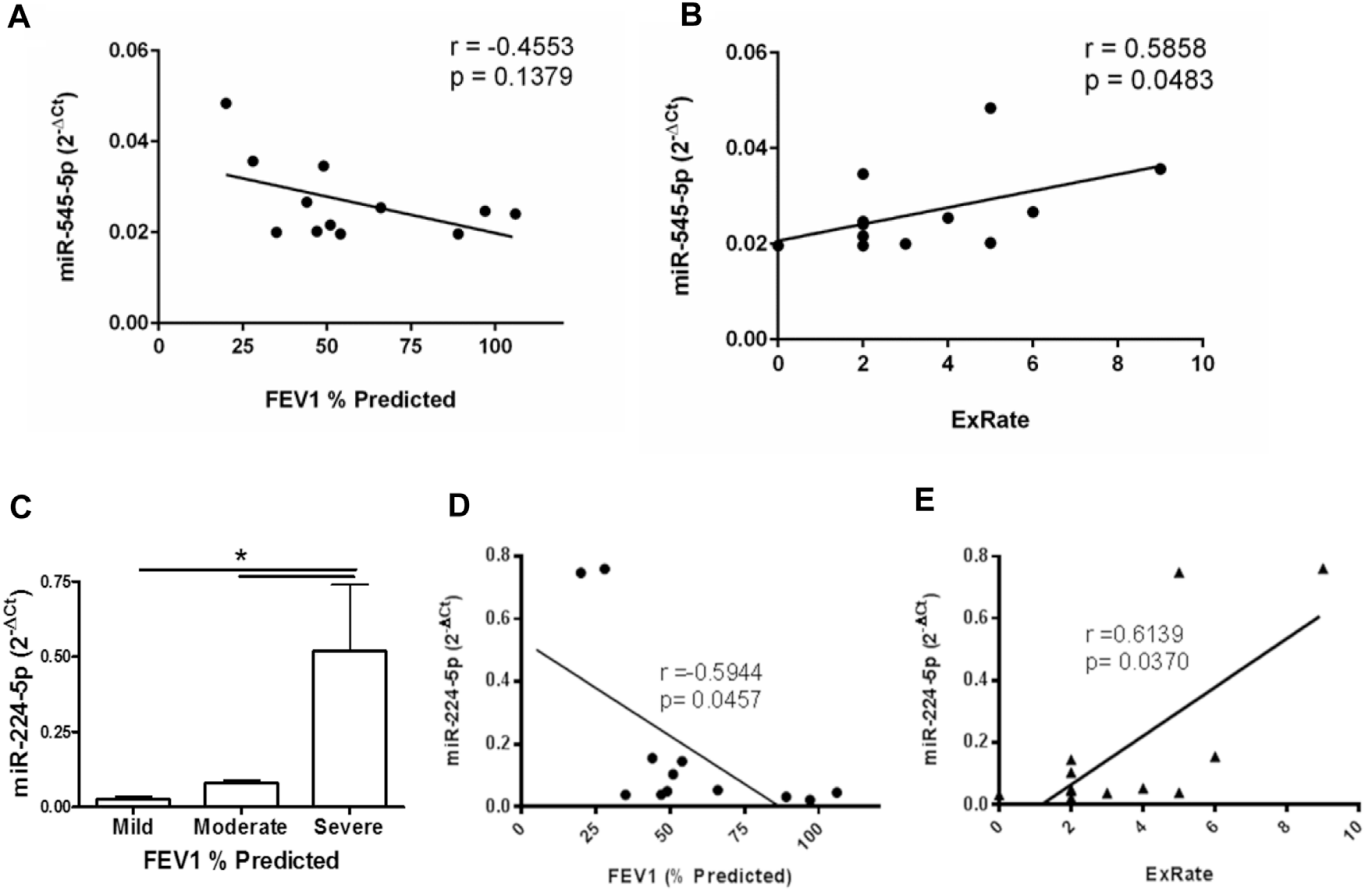
Correlation between monocyte miR-545-5p, miR-224-5p, lung function and exacerbation rate in people with CF. CD14^+^ monocytes were obtained from the peripheral blood of people with CF (*n* = 12). RNA was isolated from each sample and the expression of X-chromosome located miRNA were examined by qRT-PCR array. Correlation was examined between **(A)** FEV1% predicted and miR-545-5p expression, and **(B)** exacerbation rate and miR-545-5p expression. **(C)** Data depicts the normalised expression level (2^−ΔCt^) of miR-224-5p in monocytes of people with CF, per FEV1% predicted grouping. “Mild”: FEV1 ≥80 (*n* = 3), “Moderate”: FEV1 40–79 (*n* = 6) and “Severe”: FEV1 ≤40 (*n* = 3). *p ≤ 0.05; one-way ANOVA followed by Tukey’s multiple comparisons test, versus “Severe” group. **(D)** Correlation between FEV1% predicted and miR-224-5p expression. **(E)** Correlation between exacerbation rate and miR-224-5p expression. Correlation coefficients; *r*, were determined by Spearman’s rank test and significance determined by two-tailed *t* test.

In order to examine this further, the expression of miR-545-5p or miR-224-5p and its validated target *SMAD4* in monocytes from people with CF and markers of lung function were examined by Spearman’s rank correlation analysis. To assess any relationship between the miR-224-5p validated target, *SMAD4*, in monocytes from people with CF and lung function, *SMAD4* expression was examined with respect to FEV1% predicted. *SMAD4* expression only weakly positively correlated with FEV1% predicted (*r* = 0.31) and the effect was not significant. There was no correlation between *SMAD4* and exacerbation rate (data not shown). Increasing levels of miR-545-5p were associated with decreasing lung function and increased exacerbation rate (Figures 3A,B). Although its expression was only moderately negatively correlated with FEV1% predicted (*r* = -0.4553, *p* > 0.05), the positive correlation between miR-545-5p levels and exacerbation rate was strong (r = 0.5858) and statistically significant (*p* = 0.0483).

All individuals with CF were clustered into three groups depending on lung function as measured by FEV1% predicted (Figure 3C). These were mild (FEV1 ≥80%), moderate (FEV1 40–79%) and severe (FEV1<40%). There was no significant difference in miR-224-5p expression between the “mild” and “moderate” groups. However, the expression of miR-224-5p was significantly higher in the severe versus mild (adjusted *p* value: 0.0377) and moderate (adjusted *p* value: 0.0350) groups (Figure 3C). Finally, a significant strong negative correlation (*r* = -0.5944, *p* = 0.0457) was observed between FEV1% predicted and miR-224-5p expression, indicating that low FEV1% predicted values are associated with high miR-224-5p expression (Figure 3D). Conversely, a significant strong positive correlation (*r* = 0.6139, *p* = 0.0370) between exacerbation rate and miR-224-5p expression was observed (Figure 3E) indicating that increasing numbers of exacerbations are associated with high miR-224-5p expression. Multiple linear regression was also performed to examine the relationship between clinical parameters and the combination of miR-224-5p and miR-545-5p levels, however no statistical significance was found (Supplementary Figure S4).

## Discussion

It is known that altered miRNA expression in CF bronchial brushings relates to functional changes that can contribute to CF lung disease pathophysiology. Prior to the present study, it was unknown whether expression levels of miRNA encoded on the X chromosome are associated with CF lung disease. Here we demonstrate that in people with CF, miR-224-5p expression in peripheral blood CD14^+^ monocytes inversely correlates with lung function and positively correlates with exacerbation rate. MiR-545-5p levels are also related to exacerbation rate, albeit less robustly. Collectively the data highlight miR-224-5p as a potential biomarker for CF lung decline.

We previously reported that there is clear difference in the expression of X-linked miRNAs in CF versus non-CF monocytes (McKiernan et al., 2018). Here, two of the top seven miRNAs with the highest expression in the CF group, miR-224-5p and miR-545-5p, were examined further. Expression of both X-linked miRNAs was significantly correlated with exacerbation rate in CF; for miR-224-5p there was also a clear correlation with FEV1% predicted. High levels of miR-224-5p were associated with poor lung function; specifically, low FEV1% predicted values and high numbers of exacerbations per year. In this study an exacerbation was defined as worsening of the patient’s respiratory symptoms requiring treatment with antibiotics. These results raise the possibility that this miRNA may be a biomarker for lung function decline in CF or other chronic inflammatory lung diseases. Despite the well-documented sexual dimorphisms in CF pathology, no statistically significant sex differences were observed in lung parameters in this small patient cohort, potentially due to the heterogeneous nature of CF lung disease. No individual miRNAs were differentially expressed between CF males and females, which may indicate that the increased expression of X-linked miR-224-5p and miR-545-5p in CF monocytes is a result of the same mechanisms in both sexes, i.e., increased transcription of these miRNAs on the active X chromosome (females and males) rather than escape from X chromosome inactivation in females.

Ideal biomarkers should be detectable in easily accessed biological samples, and miR-224-5p already fulfils this criterion as it can be detected in serum (Gui et al., 2011; Li et al., 2011; Vencken et al., 2015). Future studies should examine whether this miRNA, or miR-545-5p, display a similar profile in serum of people with CF. Somewhat related to this, we have recently performed miRNA expression profiling in plasma of children with CF (Mooney et al., 2020). The focus of that study was to determine whether sex differences exist in the miRNA profile between boys and girls with CF, and its major finding was that there is a significant increase in miR-885-5p in plasma of females versus males with CF under 6 years of age. There was no non-CF group in that study with which we could retrospectively compare miR-224-5p or miR-545-5p levels.

Ideozu et al. (2019) performed miRNA profiling of plasma samples from people with CF versus healthy controls and found 11 differentially expressed miRNAs, including the X-linked miR-222-3p. This miRNA was not selected by the authors for further validation by qPCR but it would be interesting to see if its expression was significantly altered in a larger cohort, including equal numbers of male/female samples in both the CF and non-CF groups, and if there are any correlations with clinical parameters. A recent study by Stachowiak et al. (2020) reported a number of miRNAs from airway samples to show correlation with CF pulmonary exacerbation parameters, one of which was an X-linked miRNA, miR-223-3p. Increased levels of miR-223-3p were found in exhaled breath condensate and sputum during pulmonary exacerbation with concurrent *Aspergillus* infection.

*SMAD4* is a validated target of miR-224-5p. Its levels have been shown to be significantly decreased in the CF subjects in our study and to weakly inversely correlate with miR-224-5p levels (McKiernan et al., 2018). Here we observed no strong or significant correlation between *SMAD4* expression in CF monocytes and FEV1% predicted or exacerbation rate.

Overall this pilot study reveals new insights into the expression of X-linked miRNA in CF peripheral blood monocytes as potential biomarkers for CF lung function decline. The number of samples used here, whilst appropriate for array-based studies, is very small therefore, we encourage others to replicate our clinical observations in larger CF cohorts which may be available to them. Furthermore, future validation studies should expand to include samples from patients with different CF genotypes e.g., homozygous p. Phe508del and other mutations such as G551D and R117H. The effects of CFTR modulator therapy on miRNA profiles, particularly miR-224-5p and miR-545-5p, should also be evaluated. In addition it would be interesting to study the evolution of miRNA in patients before, during, and after an exacerbation. In summary, an altered X-linked miRNA profile is evident in people with CF, and some of these miRNAs; in particular miR-224-5p, correlate with clinical lung disease. Large cohort studies are warranted to confirm the utility of miR-224-5p or miR-545-5p as potential clinical biomarkers for CF disease progression.

## Materials and Methods

### Isolation of CD14^+^ Monocytes

Blood was mixed with an equal volume of 0.9% NaCl (1× saline) and layered over Lymphoprep (Axis Shield). Density gradient centrifugation was carried out at 800 *× g* for 10 min, the mononuclear cell band was aspirated, washed in Hank’s Balanced Salt Solution (Lonza, BE10-543F), and monocytes were purified using the EasySep^®^ Human CD14 Selection Cocktail (StemCell Technologies, 18058) as per the manufacturer’s protocol. This kit yields up to 97% purity of monocytes. When first establishing the method in the laboratory this was tested and we routinely isolated >95% monocytes.

### miScript™ Reverse Transcription for Polymerase Chain Reaction Array and Analysis

A total of 250 ng RNA extracted using the miRNeasy kit was reverse transcribed in a 1-step protocol into cDNA to use as templates for miScript PCR arrays. The reverse transcription reaction was prepared on ice then placed at 37°C for 60 min and then inactivated at 95°C for 5 min.

miRBase release 20 was utilised for identification of miRNAs located on the X chromosome. The 86 out of 118 entries in miRBase were chosen based on high confidence [sequences that have at least 10 deep sequencing reads that map to each of the 2 mature microRNA sequences (-5p and -3p)]. Each well of the custom made 96-well miScript™ PCR array plates (CMIHS02174), manufactured by SABiosciences, contained primers, reverse transcription reaction, PCR reaction and miRNA normalisation controls. Mature miRNA expression was measured with qRT-PCR using SYBR-Green based miScript PCR array according to the manufacturer’s instructions on a LightCycler^®^ 480, with pre-incubation step at 95°C for 15 min for HotStarTaq DNA Polymerase activation. The PCR cycles were i) denaturation at 95°C × 15 s, ii) annealing at 55°C × 30 s, iii) extension at 70°C × 30 s (repeated 40 times). Data analysis was performed on Ct values using SABiosciences PCR array data analysis software available at the time at http://pcrdataanalysis.sabiosciences.com/mirna. Relative expression was calculated using the 2^−ΔΔCt^ method (Livak and Schmittgen, 2001) using the most stable small RNA controls. A combination of descriptive statistics and the NormFinder Excel add-in (Andersen et al., 2004) was used to identify suitable small RNAs for normalisation of data.

### Gene Expression Analysis by Quantitative Real Time-Polymerase Chain Reaction

A total RNA of 200–1000 ng extracted using TRI Reagent were reverse transcribed into cDNA using the Quantitect^®^ Reverse Transcription Kit (Qiagen, 205313).

qRT-PCR primers were designed using Primer 3 online software (http://frodo.wi.mit.edu) and Primer-BLAST (http://www.ncbi.nlm.nih.gov/tools/primer-blast) and were obtained from MWG Eurofins Operon. GAPDH: (Fwd)- CAT GAG AAG TAT GAC AAC AGC CT, (Rvs)- AGT CCT TCC ACG ATA CCA AAG T; *β*-actin: (Fwd)- GGA CTT CGA GCA AGA GAT GG, (Rvs)- AGG AAG GAA GGC TGG AAG AG; SMAD4: (Fwd)- TGC ATT CCA GCC TCC CAT TT, (Rvs)- TGT GCA ACC TTG CTC TCT CA. Annealing temperatures were 57, 56 and 57°C respectively.

qRT-PCR was performed on a LightCycler^®^ 480 (Roche) using a SYBR Green master mix (Roche, 04,707,516,001). For 20 μl reactions, optimal concentrations (200–500 nM) of each forward and reverse primer were used. Template cDNA was used at a concentration of 10% of the total reaction volume. The qRT-PCR programme was 95°C for 5 min followed by 45 cycles of 95°C for 10 s, 57°C for 10 s (primer-dependent) and 72°C for 10 s (25 bases/second) respectively. The 2^−ΔΔCt^ method was used to quantify the expression of target genes relative to GAPDH and/or ACTB reference genes (Livak and Schmittgen, 2001). All qRT-PCR experiments included no-RTase and no-template controls.

### Statistical Analysis

Data were analysed with GraphPad Prism 4.0 software package (GraphPad Software, San Diego, CA). All data are depicted as Mean ± SEM unless otherwise stated. Specific analyses that were performed are described for each figure. Differences were considered significant at *p* ≤ 0.05.

## Data Availability

The original contributions presented in the study are included in the article/Supplementary Materials, further inquiries can be directed to the corresponding author.
