# Supplementary material for: miR-224-5p and miR-545-5p Levels Relate to Exacerbations and Lung Function in a Pilot Study of X-Linked MicroRNA Expression in Cystic Fibrosis Monocytes
Source: Front Genet. 2021 Nov 12;12:739311. doi: 10.3389/fgene.2021.739311 (PMC8633565; doi:10.3389/fgene.2021.739311)
Supplement: Supplementary file 1 [file DataSheet1.PDF]

Supplementary Figure 1

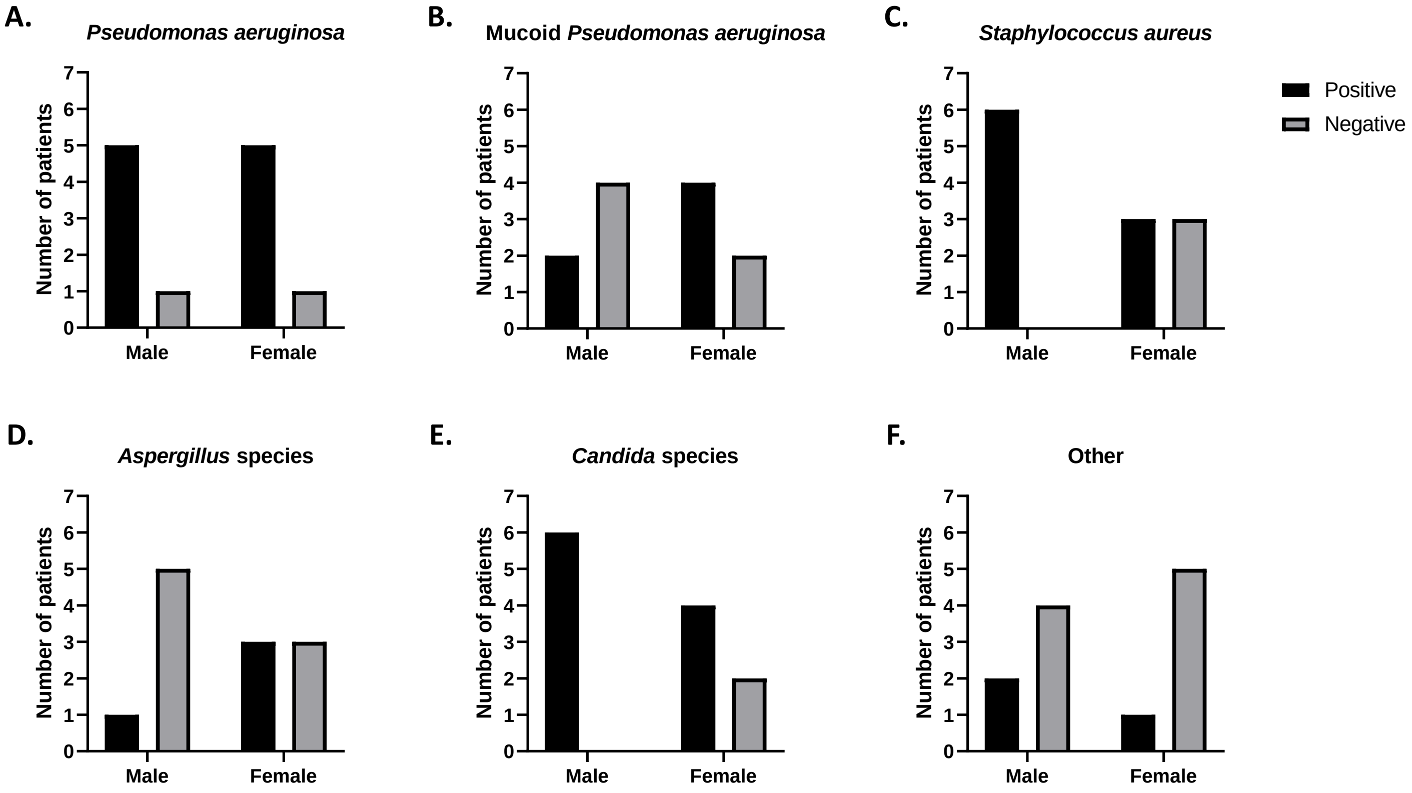

Supplementary Figure 1. The colonisation status of the CF male and CF female cohorts with (A) *Pseudomonas aeruginosa*, (B) mucoid *Pseudomonas aeruginosa*, (C) *Staphylococcus aureus*, (D) *Aspergillus* species, (E) *Candida* species, (F) Other – includes methicillin-resistant *Staphylococcus aureus*, *Burkholderia cepacia* complex and *Stenotrophomonas maltophilia* as detailed in Table 1. Data are presented as the number of patients positive or negative for infection with each specified pathogen. Contingency table analysis (Fisher's exact test) was performed in GraphPad Prism. No statistically significant difference was found between the male and female cohorts for colonisation with any of the pathogens shown.

Supplementary Figure 2

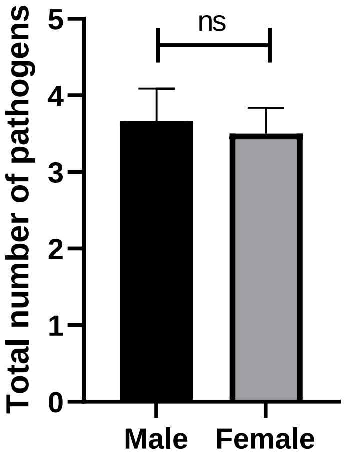

Supplementary Figure 2. The colonisation status of the CF male and CF female cohorts. Data are presented as the mean +/- SEM of the total number of pathogen species detected in each patient. Data were analysed by Mann Whitney test in GraphPad Prism. No statistical significance was found.

Supplementary Figure 3

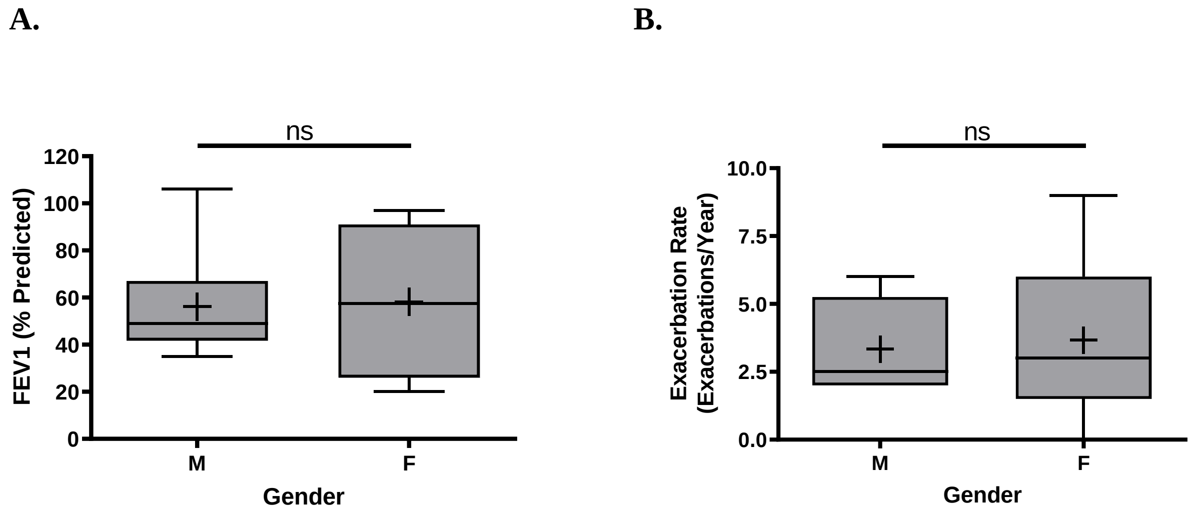

Supplementary Figure 3. Lung function and exacerbation rate of the CF cohort. (A) Box and whisker plot of FEV1 percent predicted as a determinant of lung function across males and females (n=6 each). (B) Box and whisker plot of exacerbation rate, defined as the current number of exacerbations per year, across both genders (n=6 each). Centre lines depict medians; box limits indicate the 25th and 75th percentiles; whiskers extend to minimum and maximum values and means are represented as a '+'.

Supplementary Figure 4

A.

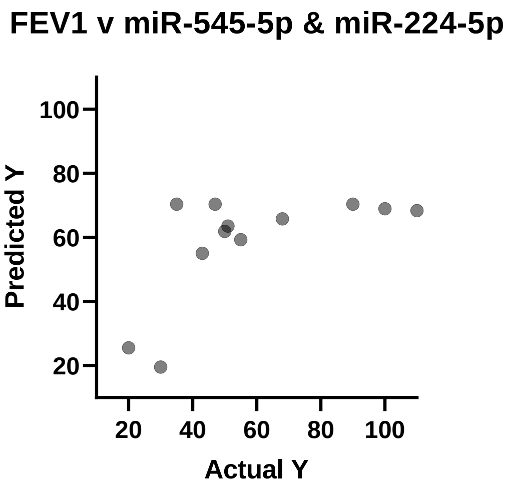

Parameter estimates	Variable	P value
β0	Intercept	0.1132
β1	miR-545-5p	0.7800
β2	miR-224-5p	0.5536
β3	miR-545-5p : miR-224-5p	0.7716

R^2 = 0.3784

B.

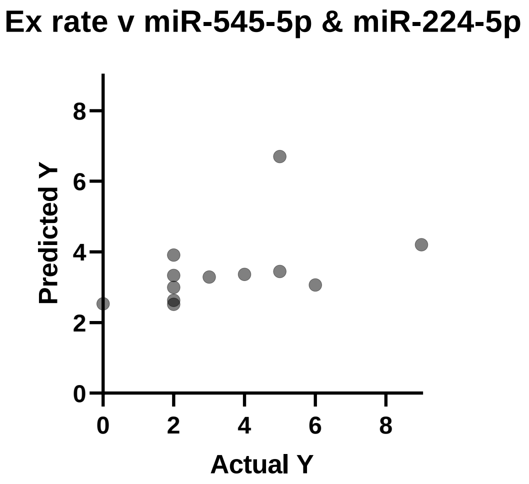

Parameter estimates	Variable	P value
β0	Intercept	0.7404
β1	miR-545-5p	0.1907
β2	miR-224-5p	0.5426
β3	miR-545-5p : miR-224-5p	0.4972

R^2 = 0.2173

Supplementary Figure 4. Multiple linear regression of clinical parameters versus miR-545-5p and miR-224-5p. CD14+ monocytes were obtained from the peripheral blood of people with CF (n=12). RNA was isolated and miRNA expression measured by qRT-PCR. Multiple linear regression of (A) FEV1 % predicted and (B) exacerbation rate versus miR-545-5p and miR-224-5p was performed using GraphPad Prism. No statistical significance was found.

Supplementary Table 1. Top seven differentially expressed X-linked miRNAs in CF vs. non-CF monocytes

X miRNA	FC* increase vs. non-CF	*P* value
miR-224-5p	3.0-fold	0.1336
miR-452-5p	1.9-fold	0.1242
miR-450b-5p	1.6-fold	0.0028
miR-542-3p	1.6-fold	0.0021
miR-450a-5p	1.6-fold	0.0043
miR-424-5p	1.6-fold	0.0046
miR-545-5p	1.5-fold	0.0245

*FC, fold change.

Supplementary Table 2 Microarray data of All non-CF versus All CF.

miRNA ID	Well	AVG ΔC_t (Ct(GOI) - Ave Ct (HKG))		$2^{-\Delta C_t}$		Fold Difference	T-TEST	Fold Up- or Down-Regulation
		Test Sample	Control Sample	Test Sample	Control Sample	Test Sample /Control Sample	p value	Test Sample /Control Sample
hsa-miR-224-5p	E04	10.19	11.79	8.6E-04	2.8E-04	3.03	0.133644	3.03
hsa-miR-452-5p	E07	13.11	14.01	1.1E-04	6.1E-05	1.87	0.124177	1.87
hsa-miR-450b-5p	E06	7.29	7.99	6.4E-03	3.9E-03	1.63	0.002844	1.63
hsa-miR-542-3p	C05	6.79	7.48	9.0E-03	5.6E-03	1.62	0.002128	1.62
hsa-miR-450a-5p	B06	4.74	5.39	3.7E-02	2.4E-02	1.57	0.004276	1.57
hsa-miR-424-5p	B05	1.58	2.22	3.3E-01	2.1E-01	1.55	0.004602	1.55
hsa-miR-545-5p	A04	8.41	9.03	2.9E-03	1.9E-03	1.54	0.024484	1.54
hsa-miR-221-5p	E01	7.56	8.13	5.3E-03	3.6E-03	1.49	0.003070	1.49
hsa-miR-503-5p	B09	6.47	6.98	1.1E-02	7.9E-03	1.43	0.014216	1.43
hsa-miR-222-3p	A10	2.53	3.01	1.7E-01	1.2E-01	1.40	0.001201	1.40
hsa-miR-221-3p	D12	2.02	2.50	2.5E-01	1.8E-01	1.40	0.015019	1.40
hsa-miR-374b-5p	B04	2.54	2.99	1.7E-01	1.3E-01	1.36	0.020070	1.36
hsa-miR-4329	G06	14.18	14.62	5.4E-05	4.0E-05	1.36	0.017704	1.36
hsa-miR-374a-5p	B03	1.90	2.33	2.7E-01	2.0E-01	1.35	0.031799	1.35
hsa-miR-98-5p	D03	3.96	4.38	6.4E-02	4.8E-02	1.34	0.019929	1.34
hsa-miR-19b-3p	D11	-0.72	-0.30	1.6E+00	1.2E+00	1.33	0.047560	1.33
hsa-miR-421	G04	5.93	6.31	1.6E-02	1.3E-02	1.30	0.014172	1.30
hsa-miR-4328	G05	14.24	14.62	5.2E-05	4.0E-05	1.30	0.063104	1.30
hsa-miR-548m	G08	14.41	14.78	4.6E-05	3.5E-05	1.29	0.053970	1.29
hsa-miR-20b-5p	A07	1.31	1.67	4.0E-01	3.1E-01	1.29	0.070595	1.29
hsa-miR-513c-5p	C04	14.42	14.78	4.6E-05	3.5E-05	1.28	0.058515	1.28
hsa-miR-92a-2-5p	D01	14.42	14.78	4.6E-05	3.5E-05	1.28	0.058515	1.28
hsa-miR-513b-5p	F05	14.42	14.78	4.6E-05	3.5E-05	1.28	0.058515	1.28
hsa-miR-325	G02	14.42	14.78	4.6E-05	3.5E-05	1.28	0.058515	1.28
hsa-miR-384	G03	14.42	14.78	4.6E-05	3.5E-05	1.28	0.058515	1.28
hsa-let-7f-5p	A01	-0.57	-0.21	1.5E+00	1.2E+00	1.28	0.017626	1.28
hsa-miR-891b	G11	14.41	14.77	4.6E-05	3.6E-05	1.28	0.059977	1.28
hsa-miR-545-3p	C06	8.28	8.64	3.2E-03	2.5E-03	1.28	0.222181	1.28
hsa-miR-223-3p	E02	-5.18	-4.83	3.6E+01	2.8E+01	1.27	0.040610	1.27
hsa-miR-892a	G12	14.40	14.74	4.6E-05	3.6E-05	1.27	0.054094	1.27
hsa-miR-513a-3p	F04	14.42	14.77	4.6E-05	3.6E-05	1.27	0.062628	1.27
hsa-miR-651-5p	C08	9.33	9.67	1.6E-03	1.2E-03	1.26	0.073737	1.26
hsa-miR-18b-5p	D10	1.59	1.91	3.3E-01	2.7E-01	1.25	0.249144	1.25
hsa-miR-507	G07	14.42	14.73	4.6E-05	3.7E-05	1.23	0.079725	1.23
hsa-miR-505-3p	F02	5.03	5.33	3.1E-02	2.5E-02	1.22	0.023551	1.22
hsa-miR-660-5p	C10	3.41	3.69	9.4E-02	7.8E-02	1.21	0.037066	1.21
hsa-miR-501-5p	E10	7.33	7.61	6.2E-03	5.1E-03	1.21	0.031148	1.21
hsa-miR-3202	F12	14.17	14.44	5.4E-05	4.5E-05	1.20	0.150589	1.20
hsa-miR-502-5p	E12	7.21	7.48	6.7E-03	5.6E-03	1.20	0.001082	1.20
hsa-miR-223-5p	E03	3.78	4.05	7.3E-02	6.1E-02	1.20	0.047579	1.20
hsa-miR-508-3p	B12	14.40	14.66	4.6E-05	3.9E-05	1.20	0.155917	1.20
hsa-miR-501-3p	E11	7.05	7.31	7.5E-03	6.3E-03	1.19	0.008157	1.19
hsa-miR-892b	H01	14.33	14.58	4.8E-05	4.1E-05	1.19	0.146699	1.19

hsa-miR-652-3p	C09	3.57	3.80	8.4E-02	7.2E-02	1.18	0.039197	1.18
hsa-miR-766-3p	C11	6.11	6.34	1.5E-02	1.2E-02	1.17	0.251756	1.17
hsa-miR-513a-5p	F03	13.98	14.21	6.2E-05	5.3E-05	1.17	0.816560	1.17
hsa-miR-361-3p	A11	5.62	5.84	2.0E-02	1.7E-02	1.17	0.142571	1.17
hsa-miR-362-5p	A12	5.75	5.96	1.9E-02	1.6E-02	1.16	0.012965	1.16
hsa-miR-92a-3p	D02	-0.06	0.16	1.0E+00	9.0E-01	1.16	0.057694	1.16
hsa-miR-532-3p	F07	4.70	4.91	3.8E-02	3.3E-02	1.16	0.072786	1.16
hsa-miR-1264	D05	13.95	14.15	6.3E-05	5.5E-05	1.15	0.587252	1.15
hsa-miR-532-5p	F06	5.34	5.54	2.5E-02	2.1E-02	1.15	0.082156	1.15
hsa-miR-1321	D07	13.66	13.86	7.7E-05	6.7E-05	1.14	0.638411	1.14
hsa-miR-500a-5p	B07	5.96	6.15	1.6E-02	1.4E-02	1.14	0.059834	1.14
hsa-miR-500b-5p	E09	5.58	5.75	2.1E-02	1.9E-02	1.13	0.034946	1.13
hsa-miR-676-3p	F09	14.16	14.34	5.4E-05	4.8E-05	1.13	0.829069	1.13
hsa-miR-500a-3p	B08	6.82	6.99	8.8E-03	7.9E-03	1.12	0.022935	1.12
hsa-miR-362-3p	B01	4.51	4.67	4.4E-02	3.9E-02	1.12	0.248627	1.12
SNORD72	H06	2.65	2.81	1.6E-01	1.4E-01	1.12	0.686650	1.12
hsa-miR-374c-5p	E05	12.38	12.53	1.9E-04	1.7E-04	1.11	0.775866	1.11
hsa-miR-548am-3p	C07	9.90	10.05	1.0E-03	9.5E-04	1.11	0.477586	1.11
hsa-miR-502-3p	F01	6.19	6.32	1.4E-02	1.2E-02	1.10	0.044767	1.10
hsa-miR-509-3-5p	C02	14.42	14.56	4.6E-05	4.2E-05	1.10	0.381440	1.10
SNORD68	H05	-1.33	-1.19	2.5E+00	2.3E+00	1.10	0.170926	1.10
hsa-miR-1184	D04	11.38	11.51	3.8E-04	3.4E-04	1.09	0.463496	1.09
hsa-miR-509-3p	C01	14.38	14.50	4.7E-05	4.3E-05	1.09	0.647291	1.09
hsa-miR-105-3p	A03	13.59	13.71	8.1E-05	7.5E-05	1.08	0.902025	1.08
hsa-miR-718	G09	6.00	6.11	1.6E-02	1.4E-02	1.08	0.546947	1.08
hsa-miR-1468-5p	D08	12.97	13.08	1.2E-04	1.2E-04	1.08	0.917342	1.08
hsa-miR-363-3p	B02	5.30	5.38	2.5E-02	2.4E-02	1.06	0.741822	1.06
hsa-miR-1298-5p	D06	13.62	13.71	7.9E-05	7.5E-05	1.06	0.790465	1.06
hsa-miR-188-5p	A05	5.99	6.07	1.6E-02	1.5E-02	1.06	0.271312	1.06
RNU6-2	H03	-1.45	-1.38	2.7E+00	2.6E+00	1.05	0.462856	1.05
hsa-miR-105-5p	A02	14.27	14.32	5.1E-05	4.9E-05	1.04	0.939014	1.04
hsa-miR-320d	G01	11.18	11.22	4.3E-04	4.2E-04	1.02	0.567939	1.02
hsa-miR-20b-3p	A08	10.28	10.31	8.0E-04	7.9E-04	1.02	0.999321	1.02
SNORD95	H07	0.40	0.40	7.6E-01	7.6E-01	1.01	0.869899	1.01
SNORD61	H04	0.34	0.35	7.9E-01	7.8E-01	1.00	0.804956	1.00
hsa-miR-890	G10	14.27	14.27	5.1E-05	5.1E-05	1.00	0.595744	-1.00
hsa-miR-2114-5p	A09	13.98	13.91	6.2E-05	6.5E-05	0.96	0.647197	-1.04
hsa-miR-1912	F11	11.00	10.93	4.9E-04	5.1E-04	0.95	0.563077	-1.05
hsa-miR-506-3p	B11	12.49	12.41	1.7E-04	1.8E-04	0.95	0.909791	-1.06
SNORD96A	H08	0.71	0.62	6.1E-01	6.5E-01	0.94	0.353824	-1.06
hsa-miR-934	H02	13.97	13.79	6.2E-05	7.1E-05	0.88	0.363370	-1.14
hsa-miR-891a-5p	F10	9.14	8.95	1.8E-03	2.0E-03	0.88	0.429121	-1.14
hsa-miR-767-5p	C12	13.06	12.84	1.2E-04	1.4E-04	0.86	0.517629	-1.16
hsa-miR-1587	D09	7.49	7.25	5.5E-03	6.6E-03	0.84	0.400406	-1.19
hsa-miR-504-5p	B10	13.07	12.81	1.2E-04	1.4E-04	0.83	0.862967	-1.20
hsa-miR-510-5p	C03	12.41	12.15	1.8E-04	2.2E-04	0.83	0.287387	-1.20
hsa-miR-1911-5p	A06	13.17	12.86	1.1E-04	1.3E-04	0.81	0.337529	-1.24
hsa-miR-4769-5p	E08	11.89	11.51	2.6E-04	3.4E-04	0.77	0.269987	-1.30
hsa-miR-548aj-3p	F08	14.06	13.58	5.9E-05	8.2E-05	0.72	0.295959	-1.39
